# Haemodynamic responses to small muscle mass exercise before and after lung transplantation in end‐stage chronic obstructive pulmonary disease

**DOI:** 10.1113/EP093645

**Published:** 2026-07-14

**Authors:** Camilla Koch Ryrsø, Thomas Kromann Lund, Pia Thaning, Lasse Gliemann, Michael Perch, Jacob Peter Hartmann, Ida Kær Thorsen, Gregers Winding Munch, Stefan P. Mortensen, Ylva Hellsten, Ronan M. G. Berg, Ulrik Winning Iepsen

**Affiliations:** ^1^ Centre for Physical Activity Research Copenhagen University Hospital–Rigshospitalet Copenhagen Denmark; ^2^ Department of Cardiology, Section for Lung Transplantation Copenhagen University Hospital–Rigshospitalet Copenhagen Denmark; ^3^ Department of Respiratory Medicine University Hospital Hvidovre Hvidovre Denmark; ^4^ Department of Nutrition, Exercise and Sports, The August Krogh Section for Human Physiology University of Copenhagen Copenhagen Denmark; ^5^ Department of Clinical Medicine University of Copenhagen Copenhagen Denmark; ^6^ Department of Biomedical Sciences Faculty of Health and Medical Sciences University of Copenhagen Copenhagen Denmark; ^7^ Department of Clinical Physiology and Nuclear Medicine Copenhagen University Hospital–Rigshospitalet Copenhagen Denmark; ^8^ Department of Cardiovascular and Renal Research University of Southern Denmark Odense Denmark; ^9^ Department of Anaesthesia and Intensive Care Copenhagen University Hospital–Hvidovre Copenhagen Denmark

**Keywords:** exercise, hypoxia, microcirculation, respiratory insufficiency, vascular remodelling

## Abstract

Cardiovascular control during exercise is impaired in chronic obstructive pulmonary disease (COPD). The central cardiopulmonary pathologies in COPD might drive the local muscle vascular abnormalities. We hypothesised that contracting muscle perfusion and O_2_ utilisation improve after lung transplantation (LTx) in COPD. Ten patients with end‐stage COPD were tested on the LTx waiting list (pre‐LTx) and again 6 months after LTx (post‐LTx). We measured leg blood flow (${\dot{Q}}_{\mathrm{leg}}$, main outcome) during submaximal one‐leg knee‐extensor exercise (KEE), and femoral arterial–venous blood samples were obtained for the determination of O_2_ kinetics across the leg and vasoactive compounds. Cardiac output and stroke volume in response to KEE peak workload (WL_peak_), walking distance and physical activity (triaxial accelerometry) were also assessed. Lung function (one single and nine double LTx), walking distance and physical activity increased post‐LTx, whereas leg lean mass and WL_peak_ remained unchanged. Arterial oxygen partial pressure decreased in response to KEE pre‐LTx but was maintained at resting levels post‐LTx. Working muscle capillary O_2_ tension and saturation were also higher post‐LTx, with no difference in muscle O_2_ conductance or extraction. We found no changes in ${\dot{Q}}_{\mathrm{leg}}$ during submaximal KEE (pre‐LTx: 1026 ± 278 vs. post‐LTx: 941 ± 186 mL min^−1^, *P* = 0.49); mean arterial pressure and circulating pro‐brain natriuretic peptide levels were lower post‐LTx and cardiac output higher. Cardiac output (pre‐LTx: 6.1 ± 0.4 vs. post‐LTx: 8.3 ± 0.5 L min^−1^, *P* = 0.0001) and stroke volume responses to WL_peak_ were also higher post‐LTx. LTx did not affect muscle blood flow regulation during moderate‐intensity KEE in end‐stage COPD and seemed to be disconnected from the augmented cardiac output responses to exercise.

## Abstract (highlights)

- **What is the central question of this study?**
  Are haemodynamic responses to small muscle mass exercise adaptive in hypoxaemic end‐stage chronic obstructive pulmonary disease (COPD) patients followed to near‐normalisation of pulmonary function 6 months after lung transplantation?
- **What is the main finding and its importance?**
  Lung transplantation in COPD did not affect the leg muscle blood flow responses to small muscle mass exercise, although both cardiac output and stroke volume increased and circulating pro‐brain natriuretic peptide levels decreased. Thus, there might be a disconnection between central and peripheral haemodynamic adaptations to normalisation of pulmonary function in COPD.

## 1 INTRODUCTION

Chronic obstructive pulmonary disease (COPD) is accompanied by muscle dysfunction, which especially affects the leg muscles and includes loss of muscle mass and function, leading to reduced ambulatory capacity, quality of life and survival (Agustí et al., 2023; Maltais et al., 2014). Although the pathogenesis of limb muscle dysfunction remains debated, factors such as physical inactivity, inflammation, smoking, medication and chronic tissue hypoxia might contribute to muscle dysfunction in COPD (Debevec et al., 2018; Iepsen & Pedersen, 2020; Jaitovich & Barreiro, 2018; van Bakel et al., 2021).

The local haemodynamic consequences of the COPD‐related muscle pathologies include a diminished leg muscle blood flow, which impacts transport of O_2_ to the exercising muscle and might attenuate peripheral exercise capacity (Broxterman et al., 2020, 2021; Iepsen et al., 2017). Patients with COPD also exhibit an impaired central haemodynamic response to exercise with lower cardiac output (CO) and stroke volume (SV) in comparison to healthy subjects, probably attributable to airway obstruction, resulting in hyperinflation and mechanical suppression of cardiac function (Smith et al., 2019). Thus, both central and peripheral haemodynamic limitations to exercise are present with COPD, but abnormal neural control of the circulation might also play a role (i.e., the exercise pressor reflex) (Iepsen et al., 2021). Muscle O_2_ transport can be studied with minimal cardiopulmonary impact in COPD by activating a small muscle mass at an intensity below the ventilatory threshold (Richardson et al., 1999, 2004) through low‐intensity one‐leg knee‐extensor exercise (KEE). This model permits the determination of local muscle O_2_ delivery and utilisation during dynamic exercise (Andersen et al., 1985). In patients with end‐stage COPD, lung transplantation (LTx) is an established treatment option aiming to improve quality of life and survival (Leard et al., 2021; Verleden & Gottlieb, 2023). Although limb muscle dysfunction after LTx has been reported previously (Evans et al., 1997; Tirdel et al., 1998; Wang et al., 1999), studies were not prospective and contained no information on exercising haemodynamic adaptations, such as muscle perfusion or O_2_ utilisation. Moreover, the evidence of persistent muscle dysfunction after LTx has been based on a mixed population of LTx recipients (e.g., cystic fibrosis, interstitial lung disease) and not only COPD.

In this prospective study, therefore, we focused solely on patients with end‐stage COPD undergoing LTx to test whether the integrative haemodynamic response to submaximal KEE adapted to the restored lung function. We measured peripheral and central haemodynamic responses to exercise before LTx (pre‐LTx) and 6 months after LTx (post‐LTx). Leg muscle blood flow (${\dot{Q}}_{\mathrm{leg}}$), O_2_ utilisation and local muscle release of vasoactive compounds during submaximal KEE were the outcomes used as measures of peripheral haemodynamics. Central haemodynamics [CO, SV and pro‐brain natriuretic peptide (pro‐BNP)] were recorded during both submaximal and maximal KEE, and physical activity, walking distance and leg muscle mass and endurance were also tested pre‐LTx and post‐LTx. We hypothesised that central haemodynamic responses to KEE would improve post‐LTx, owing to less hyperinflation and mechanical compression of cardiac function, and that would affect the muscle blood flow regulation towards higher ${\dot{Q}}_{\mathrm{leg}}$.

## 2 MATERIALS AND METHODS

### 2.1 Ethical approval

The study was approved by the Ethical Committee of the Capital Region of Copenhagen (H‐17039327) and registered at ClinicalTrials.gov (NCT03548857). The study conformed to the most recent version of the *Declaration of Helsinki*, except for registration in a database, and the findings are reported in accordance with the STROBE guideline (von Elm et al., 2008). All participants provided oral and written informed consent.

Patients or the public were not involved in the design, conduct, reporting or dissemination plans of our research. No generative artificial intelligence tools were used in the preparation of this manuscript.

### 2.2 Study design and patients

Ten patients with end‐stage COPD were included when deemed eligible for the LTx waiting list. Patients were tested on two separate experimental days, pre‐LTx and post‐LTx. Patients were recruited from the Department of Cardiology, Section for LTx at Copenhagen University Hospital − Rigshospitalet, Denmark. Patients were aged ≥18 years with end‐stage COPD with or without alpha‐1 antitrypsin deficiency but without severe pulmonary arterial hypertension (pulmonary capillary wedge pressure 13.1 ± 5.4 mmHg, right atrial pressure 7.6 ± 3.5 mmHg, pulmonary vascular resistance 2.1 (25th–75th quartile, 1.0–4.5) wood units, dyn s cm^−5^, and resting CO 5.9 ± 1.7 L min^−1^). Exclusion criteria were re‐transplantation, multi‐organ transplantation, positive crossmatching, and anticoagulant medication pre‐LTx (except acetylsalicylic acid 75 mg day^−1^). Supplementary O_2_ therapy via a nasal cannula and other medications were continued throughout the experiments to ensure safety.

### 2.3 Experimental procedures

On the first experimental day, the patients visited the laboratory to become familiar with the KEE model. They performed: (1) an incremental KEE test to determine one‐leg peak workload (WL_peak_); (2) 6 min walk test (6MWT); (3) whole‐body dual‐energy X‐ray absorptiometry scanning (Lunar Prodigy Advance, GE Healthcare, WI, USA); (4) blood sample to assess systemic inflammation; (5) COPD assessment test; and (6) were administered with the accelerometer during the visit, and the assessment of physical activity was for the subsequent 7 days. During the incremental KEE test, CO, SV and mean arterial pressure (MAP) were measured continuously using finger photoplethysmography (Finometer, Finapres Medical Systems, The Netherlands) on the middle finger of the non‐dominant hand. Recordings were made through a data‐acquisition system (PowerLab 16/30, ADInstruments, Bella Vista, NSW, Australia) and later analysed (LabChart, ADInstruments, UK). CO, SV and MAP were recorded at sitting baseline for 5 min, and the last 30 s were averaged during each workload increment.

Physical activity was assessed using accelerometers (Axivity AX3, Newcastle, UK). Set‐up, download and outcome definitions have been described previously (Thorsen et al., 2022); data reduction and generation of physical activity outcomes were performed using the PhysAccel package (University of Southern Denmark) in R v.4.3.0. The accelerometers were attached to the patient's thigh and back and worn for 7 days consecutively, pre‐LTx and post‐LTx.

On the second experimental day, patients refrained from caffeine, alcohol and exercise for 24 h before the tests. After local anaesthesia (lignocaine 2%), a catheter was placed in the femoral vein of the experimental leg and the brachial artery for blood pressure monitoring and blood sampling.

After 30 min of supine rest, the patients were seated in the knee‐extensor ergometer and performed 3 × 4 min of KEE (60 kicks min^−1^) at 0 W, 10% and 20% of WL_peak_ determined at visit 1, separated by 15 min of rest. The workloads were designed to ensure that participants could complete the protocol, and we anticipated very low WL_peak_ before LTx. Thus, based on our previous experience with moderate‐to‐severe COPD, we decided to use unloaded exercise (0 W) as the absolute workload and 10%, and 20% WL_peak_ as relative workloads. Leg haemodynamics were evaluated, and arteriovenous blood samples were collected at baseline and during KEE (after 2 and 4 min). Leg blood flow could not be obtained in 2 of the 10 participants owing to safety issues with anticoagulative medicine prescribed post‐LTx, and they participated only in the non‐invasive tests.

### 2.4 Leg haemodynamics

Femoral arterial leg blood flow (${\dot{Q}}_{\mathrm{leg}}$) was measured by Doppler ultrasound (LOGIQ E5, GE Healthcare, WI, USA) equipped with a linear probe (9 MHz) (Iepsen et al., 2017). The site of measurement in the common femoral artery was distal to the inguinal ligament but above the bifurcation into the superficial and profound femoral branch to avoid turbulence from the bifurcation. Recordings were obtained at an intonation angle of <60° (Iepsen et al., 2017). The sample volume was maximised according to the width of the vessel without interference with the walls. Arterial diameter was determined during systole for each Doppler measurement from the arterial B‐mode images with the transducer parallel to the vessel and continuously recorded and averaged over 8–16 heart cycles. Intravascular blood pressures were monitored with pressure transducers (Pressure Monitoring Kit, Baxter, IL, USA) positioned at the level of the femoral artery (Iepsen et al., 2017). The recording was made via a data acquisition system (PowerLab 16/30, ADInstruments, Bella Vista, NSW, Australia) for later analysis (LabChart, ADInstruments, UK). At time points when leg haemodynamics were measured (${\dot{Q}}_{\mathrm{leg}}$ and MAP), arterial and venous blood samples were obtained simultaneously from the catheters for determination of O_2_ variables, and blood gases were analysed immediately (ABL825, Radiometer, Copenhagen, Denmark).

### 2.5 Calculations

Calculations were performed as previously described (Hartmann et al., 2022). Whole‐blood O_2_ concentration ($C_{xO_{2}}$) was calculated as:

$$C_{xO_{2}}=\mathrm{Hb}\times S_{xO_{2}}+\alpha O_{2}+P_{xO_{2}}$$

where *S* is the O_2_ saturation of haemoglobin (fraction), and *P* is the partial pressure of O_2_ (in kilopascals); x is arterial (a), femoral venous (v) or capillary (c) blood; Hb (in millimoles per litre) is the haemoglobin concentration; and α is the solubility coefficient of O_2_ in the blood (0.01 mmol L^−1^ kPa^−1^). Oxygen delivery ($\mathrm{De}l_{O_{2}}$) to the skeletal muscle and the fractional O_2_ extraction (EO_2_) by the skeletal muscle were calculated as:

$$\;\mathrm{De}l_{O_{2}}=C_{aO_{2}}\times {\dot{Q}}_{\mathrm{leg}}$$

$$EO_{2}=\frac{C_{\left(a-v\right)O_{2}}}{C_{aO_{2}}}$$

where $C_{\left(a-v\right)O_{2}}$ is the arterial‐to‐femoral venous O_2_ concentration difference. Skeletal muscle O_2_ uptake (${\dot{V}}_{O_{2}\mathrm{sm}}$) was calculated as:

$${\dot{V}}_{O_{2}\mathrm{sm}}=\mathrm{av}D_{O_{2}}\times {\dot{Q}}_{\mathrm{leg}}$$

Leg vascular conductance (LVC) was calculated as:

$$\mathrm{LVC}=\frac{{\dot{Q}}_{\mathrm{leg}}}{\mathrm{MAP}-\mathrm{FVP}}$$

where MAP is the area under the curve over 8–16 heart cycles during invasive experiments with pulse contour analysis, and FVP is femoral venous pressure.

Assuming a mitochondrial $P_{O_{2}}$ of approximately zero, as previously reported in skeletal muscle during submaximal exercise (Richardson et al., 1995), skeletal muscle O_2_ conductance ($D_{\mathrm{sm}O_{2}}$) was calculated as:

$$D_{\mathrm{sm}O_{2}}=\frac{{\dot{V}}_{O_{2}\mathrm{sm}}}{P_{\bar{c}O_{2}}}$$

where $P_{\bar{c}O_{2}}$ is the mean capillary O_2_ tension.

As described in detail elsewhere (Dahl et al., 2020; Hartmann et al., 2022), $P_{\bar{c}O_{2}}$ was calculated on the basis of an averaged capillary O_2_ dissociation curve (ODC), defined by the capillary *P*_50_ (*P*_50cap_), which reflects the dissociation constant of haemoglobin, and the capillary Hill coefficient, *h*_cap_, which is the cooperativity, was modelled from paired arterial and femoral venous blood gas values by assuming that they fulfil the Hill equation in both vascular beds and that values measured in arterial and femoral venous blood approximate the O_2_ tension and saturation in the arterial and venous ends of the capillary, respectively:

$$\;h_{\mathrm{cap}}=\frac{\left(\frac{S_{aO_{2}}}{1-S_{aO_{2}}}\right)-\ln\left(\frac{S_{vo_{2}}}{1-S_{vo_{2}}}\right)}{\ln\left(P_{aO_{2}}\right)-\ln\left(P_{vO_{2}}\right)}$$

*P*_50cap_ was then calculated by insertion of the Hill coefficient into the Hill equation using arterial or femoral venous blood gas values.

$$P_{50\mathrm{cap}}=P_{aO_{2}}{\left(\frac{S_{aO_{2}}}{1-S_{aO_{2}}}\right)}^{-\frac{1}{h_{cap}}}=P_{vO_{2}}\times {\left(\frac{S_{vO_{2}}}{1-S_{vO_{2}}}\right)}^{-\frac{1}{h_{cap}}}$$

$P_{\bar{c}O_{2}}$, mean capillary O_2_ saturation ($S_{\bar{c}O_{2}}$) and the mean capillary O_2_ concentration ($C_{\bar{c}O_{2}}$) were then derived as outlined previously (Dahl et al., 2020; Hartmann et al., 2022). Thus, $C_{\bar{c}O_{2}}$ was assumed to decrease proportionally with the distance travelled as blood flows through the capillary from the arterial inlet to the venous outlet, such that:

$$P_{\bar{c}O_{2}}=P_{vO_{2}}+\left(P_{aO_{2}}-P_{vO_{2}}\right)\times \frac{C_{aO_{2}}-C^{\prime }}{C_{aO_{2}}-C_{vO_{2}}}$$

$$S_{\bar{c}O_{2}}=S_{vO_{2}}+\left(S_{aO_{2}}-S_{vO_{2}}\right)\times \frac{C_{aO_{2}}-C^{\prime \prime }}{C_{aO_{2}}-C_{vO_{2}}}$$

where $C^{\prime }$ and $C^{\prime \prime }$ are defined as follows:

$$C^{\prime }=\alpha O_{2}\times \frac{P_{aO_{2}}+P_{vO_{2}}}{2}+\mathrm{Hb}\times {\bar{S}}_{\mathrm{ODC}}$$

$$C^{\prime \prime }=\alpha O_{2}\times {\bar{P}}_{\mathrm{ODC}}+\mathrm{Hb}\times \frac{S_{aO_{2}}+S_{vO_{2}}}{2}$$

Here, ${\bar{S}}_{\mathrm{ODC}}$ and ${\bar{P}}_{\mathrm{ODC}}$ are the mean O_2_ saturation and tension on the ODC, which are calculated by integration of the Hill equations from the arterial inlet to the venous outlet:

$${\bar{S}}_{\mathrm{ODC}}=\frac{1}{P_{aO_{2}}-P_{vO_{2}}}\times \int_{P_{vO_{2}}}^{P_{aO_{2}}}{\left(1+{\left(\frac{P_{50\mathrm{cap}}}{P_{\mathrm{cap}O_{2}}}\right)}^{h}\right)}^{-1}dP_{\mathrm{cap}O_{2}}$$

$${\bar{P}}_{\mathrm{ODC}}=\frac{1}{S_{aO_{2}}-S_{vO_{2}}}\times \int_{S_{vO_{2}}}^{S_{aO_{2}}}P_{50\mathrm{cap}}\times {\left(\frac{S_{\mathrm{cap}O_{2}}}{1-S_{\mathrm{cap}O_{2}}}\right)}^{\frac{1}{h_{cap}}}dS_{\mathrm{cap}O_{2}}$$

### 2.6 Circulating vasoactive markers in response to exercise

The stable metabolites of NO, nitrite (${\mathrm{NO}}_{2}^{-}$) and nitrate (${\mathrm{NO}}_{3}^{-}$), collectively referred to as NO_x_, were measured using a fluorometric assay kit (Cayman Chemical, Ann Arbor, MI, USA). The stable metabolite of prostacyclin, 6‐keto‐prostaglandin F_1α_ (6‐keto‐PGF_1α_), was measured using an enzyme‐linked immunosorbent assay (ELISA) kit (Cayman Chemical, Ann Arbor, MI, USA). 8‐Isoprostane was measured using an ELISA kit (Cayman Chemical, Ann Arbor, MI, USA). Pro‐BNP was measured using an ELISA kit (Thermo‐Fisher Scientific, Waltham, MA, USA), following the manufacturer's instructions. All analyses were based on samples obtained from the femoral vein at rest and at 0 W, 10% or 20% of WL_peak_.

### 2.7 Sample size calculation

Based on previous results (Iepsen et al., 2016; Iepsen et al., 2017), we estimated that a sample of eight patients would provide 80% power to detect a mean difference in ${\dot{Q}}_{\mathrm{leg}}$ to the exercising muscle during KEE of 300 mL min^−1^ from pre‐LTx to post‐LTx, with an SD of 200 mL min^−1^ and an α‐level of 0.05. Adjusted for 20% drop‐out, 10 patients were included.

### 2.8 Statistical analysis

Baseline characteristics are summarised as means with SD or medians with 25th–75th quartiles for continuous variables and counts with percentages for categorical variables. All statistical analyses were performed using available‐case analysis with GraphPad Prism v.10.1.2. Student's paired *t*‐test was used to detect changes from pre‐LTx to post‐LTx in baseline characteristics. Model assumptions, including normality, were assessed using residual and quantile–quantile plots. Linear mixed‐effects models were used to detect differences in ${\dot{Q}}_{\mathrm{leg}}$ (and other continuous outcomes) at rest and during exercise between pre‐LTx and post‐LTx. The models included fixed effects for time (sample number), treatment (pre‐LTx vs. post‐LTx) and treatment‐by‐time interaction, with the unique patient identifier as a random effect. Given that a conditional *F*‐test (pooling) in the ANOVA models generally inflates type I error, it is recommended that the *F*‐test not be used as a global pre‐test before the *post hoc* test (Janky, 2000). Therefore, we performed *post hoc* tests irrespective of the significance of the *F*‐test. Each model was checked for model assumptions (linearity, homogeneity of variance, normality of residuals and random effects, and the absence of multicollinearity) and normality before modelling. Tukey's *post hoc* test was performed to detect differences during exercise between pre‐LTx and post‐LTx. An α‐level of <0.05 was applied.

## 3 RESULTS

Characteristics of the COPD patients are presented in Table 1. Ten patients were included on the waiting list; one underwent single LTx and nine double LTx, and all participants were tested again at 6 month follow‐up post‐LTx. As expected, symptom burden, 6 min walk distance, dynamic lung volumes and pulmonary diffusion capacity improved post‐LTx (Table 1). Total lean mass also increased post‐LTx, which was probably caused by the lung transplant itself, because we found no changes in lean mass in the upper or lower extremities. Furthermore, WL_peak_ remained unchanged post‐LTx, although daily physical activity increased (Table 1).

**TABLE 1:** Baseline characteristics and measures of exercise capacity, body composition, physical activity and clinical scores in participants with end‐stage chronic obstructive pulmonary disease before and 6 months after lung transplantation.

Parameter	Pre LTx (*n* = 10)	Post LTx (*n* = 10)	*P*‐value
Age, years	56 ± 2	–	–
Sex, female, *n* (%)	6 (60)	–	–
Supplementary O_2_, *n*	8	0	–
MRC grade	5 ± 0	–	–
Exacerbations per year	2 ± 1	–	–
≥2 exacerbations per year, *n*	5	–	–
Pack years	20 ± 17	–	–
Previous smokers, *n*	10	–	–
CAT score	27 ± 5	9 ± 5	<0.001
FEV_1_, L	0.9 ± 0.5	2.3 ± 0.7	<0.001
FEV_1_, % predicted	29.8 ± 16.5	81.1 ± 26.8	<0.001
FVC, L s^−1^	2.5 ± 1.3	2.9 ± 0.7	0.262
FEV_1_/FVC, s^−1^	35.5 ± 6.1	80.9 ± 13.3	<0.001
*D*_LCO_ adjusted for haemoglobin, mmol min^−1^ kPa^−1^	2.38 ± 1.19	5.76 ± 1.57	0.002
TLC, L	7.9 ± 1.4	5.5 ± 1.2	<0.001
RV, L	5.1 ± 1.5	2.5 ± 0.6	<0.001
*K*_CO_, mmol min^−1^ kPa^−1^ L^−1^	0.59 ± 0.31	1.35 ± 0.21	<0.001
*K*_CO_, % predicted	38.3 ± 18.0	90.2 ± 17.2	<0.001
BMI, kg m^−2^	22.4 ± 3.0	24.8 ± 6.5	0.184
Total lean mass, kg	43.1 ± 9.6	46.1 ± 12.5	0.033
Leg lean mass, kg	6.2 ± 1.4	6.7 ± 1.9	0.104
6MWD, m	258 ± 97	459 ± 135	<0.001
One‐leg WL_peak_, W	18.1 ± 5.8	22.4 ± 7.1	0.144
Walking time, min day^−1^	17 ± 15	64 ± 31	0.005
Sedentary time, min day^−1^	1321 ± 59	1252 ± 61	0.028
Light physical activity time, min day^−1^	111 ± 51	154 ± 49	0.096
Moderate‐to‐vigorous physical activity time, min day^−1^	9 ± 8	33 ± 23	0.025
Steps, number day^−1^	3199 ± 1456	5519 ± 2330	0.046
Leukocytes, ×10^9^ cells L^−1^	8.8 ± 1.8	5.4 ± 2.2	<0.001
Neutrophils, ×10^9^ cells L^−1^, ×10^9^ cells L^−1^	5.9 ± 1.8	3.8 ± 1.2	0.004
Basophils, ×10^9^ cells L^−1^	0.06 ± 0.06	0.19 ± 0.40	0.506
Eosinophils, ×10^9^ cells L^−1^	0.22 ± 0.15	0.17 ± 0.21	0.164
Lymphocytes, ×10^9^ cells L^−1^	2.0 ± 0.7	0.9 ± 0.3	0.002
Monocytes, ×10^9^ cells L^−1^	0.7 ± 0.3	0.6 ± 0.2	0.184
CRP, mg L^−1^	2.5 (1.0–6.1)	3.0 (1.0–14.0)	0.319
Urea, mmol L^−1^	5.9 ± 2.2	12.7 ± 6.8	0.053
Creatinine, µmol L^−1^	70 (64–83)	143 (71–169)	0.010
eGFR, mL min^−1−1^ 1.73 m^−2^	90 (75–90)	51 (33–83)	0.012

*Note*: Data are expressed as the mean ± SD. Student's paired *t*‐tests were used to detect differences between before and 6 months after LTx.

Abbreviations: BMI, body mass index; CAT, COPD assessment test; *D*_LCO_, diffusing capacity for carbon monoxide; FEV_1_, forced expiratory volume exhaled in 1 s; FVC, forced vital capacity; *K*_CO_, carbon monoxide transfer coefficient; LTx, lung transplantation; TLC, total lung capacity; WL_peak_, peak workload during one‐leg knee extension exercise; 6MWD, 6 min walking distance.

All LTx recipients received standard immunosuppressants at the 6 month follow‐up post‐LTx [prednisolone 5 mg, calcineurin inhibitor (ciclosporin or tacrolimus) and mycophenolate].

### 3.1 Local muscle and central haemodynamics

**FIGURE 1:**
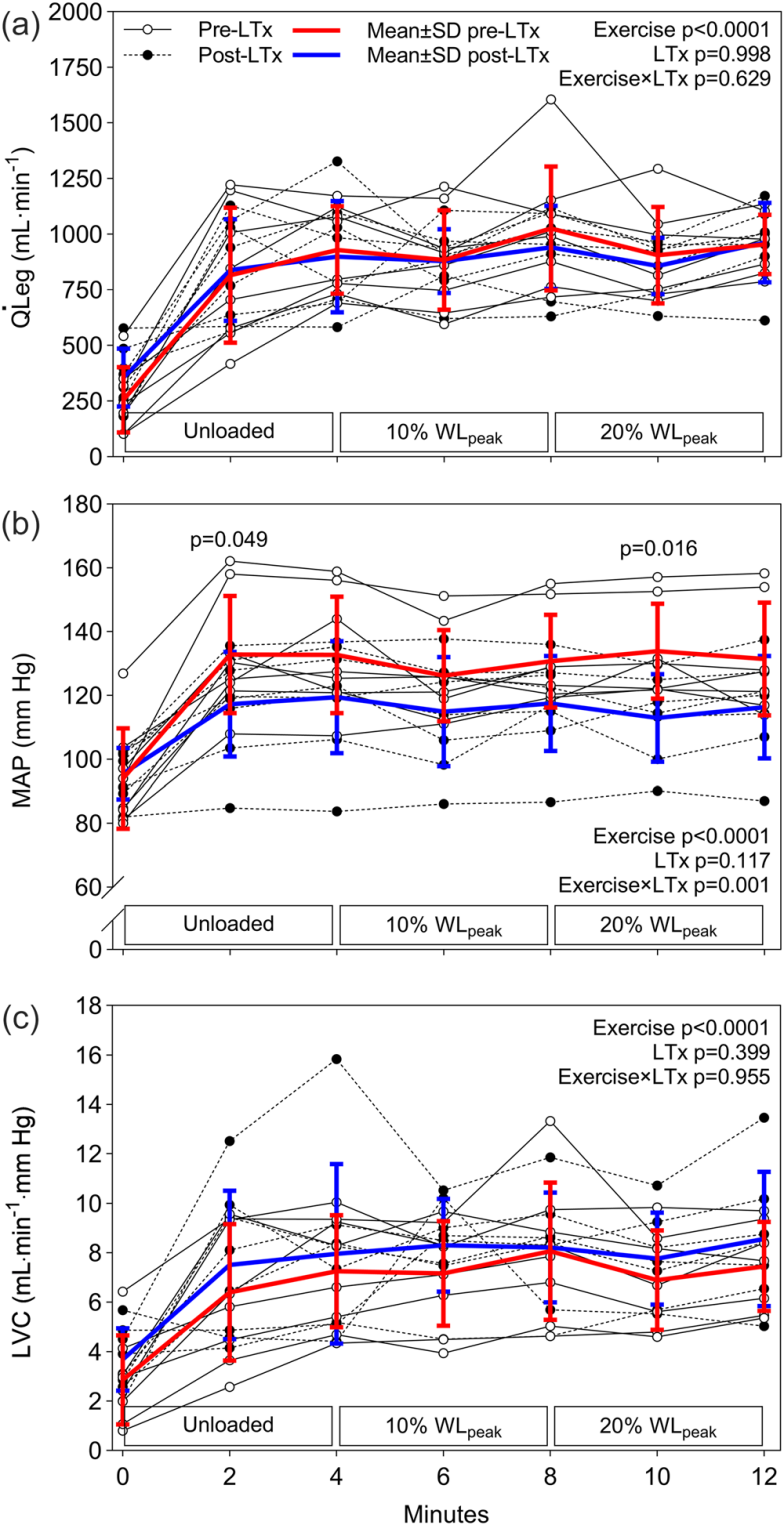
Peripheral haemodynamics during submaximal one‐leg knee‐extensor exercise before and after lung transplantation (LTx). Leg blood flow (${\dot{Q}}_{\mathrm{leg}}$, *n* = 8; a), mean arterial pressure (MAP, *n* = 8; b) and leg vascular conductance (LVC, *n* = 8; c) at rest (0 min) and during 4 min of unloaded (0 W) knee‐extensor exercise (KEE), 4 min of KEE at 10% peak workload (WL_peak_) and 4 min of KEE at 20% WL_peak_. Data are presented as individual values (pre‐LTx, open circles with continuous line; post‐LTX, filled circles with dashed line) and mean ± SD (pre‐LTx, red line; post‐LTxm blue line). A linear mixed‐effect model with Tukey's *post hoc* test was used to explore differences between pre‐LTx and post‐LTx (filled circles).

**FIGURE 2:**
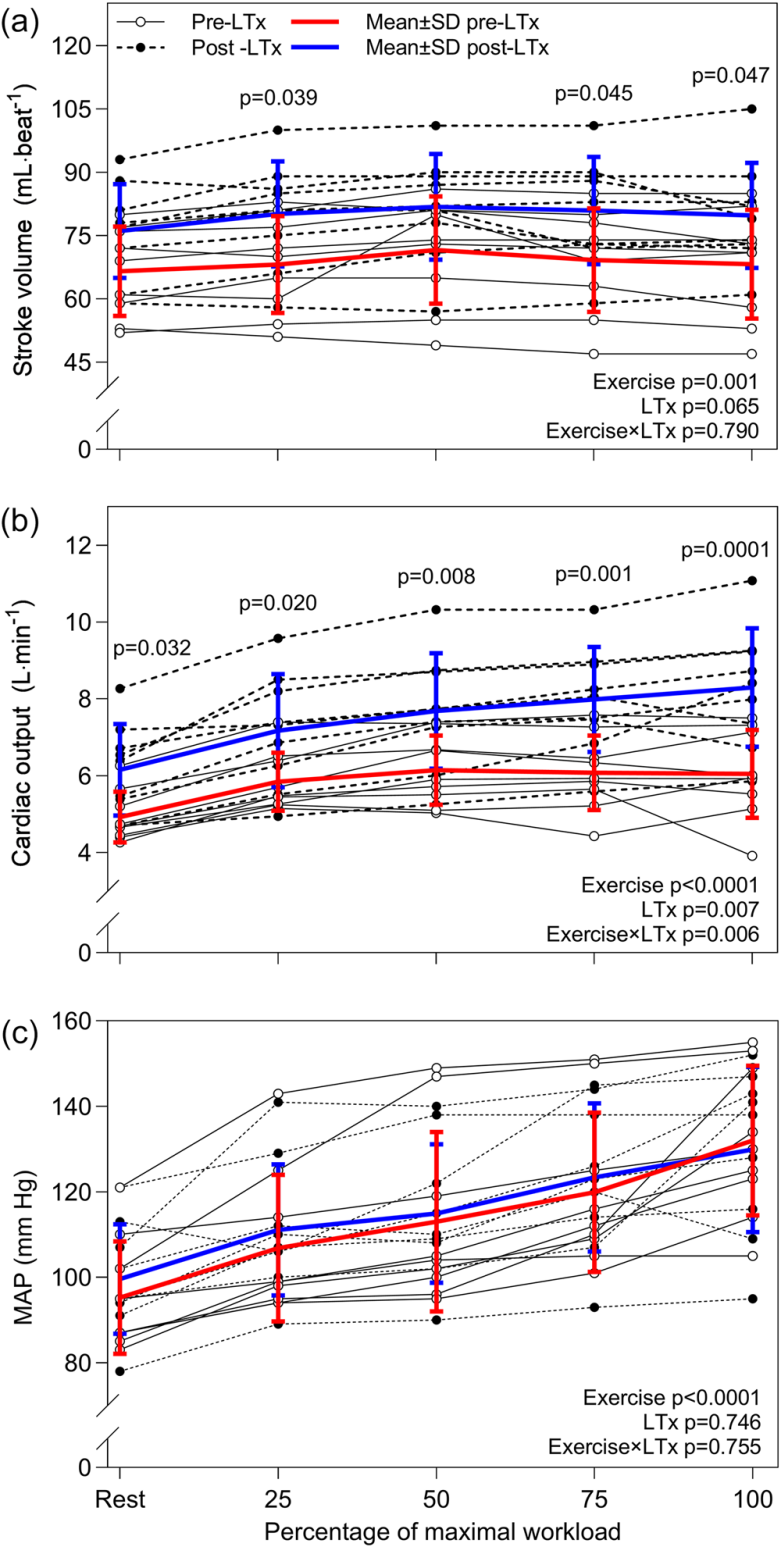
Central haemodynamic responses during incremental one‐leg knee‐extensor exercise to exhaustion before and after lung transplantation (LTx). There was no difference in maximal leg muscle workload before lung transplantation (pre‐LTx) vs. 6 months after transplantation (post‐LTx). Haemodynamic variables, including mean arterial pressure (MAP), are presented at rest and during 25%, 50%, 75% and 100% of maximal workload. Data are presented as individual values (pre‐LTx, open circles with continuous line; post‐LTX, filled circles with dashed line) and the mean ± SD (pre‐LTx, red line; post‐LTx, blue line). Comparisons were made with linear mixed models and Tukey's *post hoc* test and based on absolute values because maximal workloads were different between patients. Pre‐LTx *n* = 9 vs. post‐LTx *n* = 9 (filled circles).

Resting ${\dot{Q}}_{\mathrm{leg}}$ or LVC did not change post‐LTx (Figure 1). The ${\dot{Q}}_{\mathrm{leg}}$ and LVC increased to a similar level during unloaded KEE, with no difference between pre‐LTx and post‐LTx responses (${\dot{Q}}_{\mathrm{leg}}$, *P* = 0.802; LVC, *P* = 0.647). Likewise, at 10% or 20% WL_peak_, ${\dot{Q}}_{\mathrm{leg}}$ and LVC responses were unaltered by LTx (${\dot{Q}}_{\mathrm{leg}}$, 10% WL_peak_ *P* = 0.486, 20% WL_peak_ *P* = 0.920; LVC, 10% WL_peak_ *P* = 0.912, 20% WL_peak_ *P* = 0.388; Figure 1). In contrast, during unloaded KEE and 20% of WL_peak_, MAP responses were significantly lower post‐LTx (MAP unloaded, *P* = 0.049; MAP 20% WL_peak_, *P* = 0.016), and CO during 10% WL_peak_ was higher (pre‐LTx, 6.2 ± 1.1 vs. post‐LTx, 7.5 ± 1.3 L min^−1^, *P* = 0.048). Higher CO (rest *P* = 0.032, 25% WL_max_ *P* = 0.020, 50% WL_max_ *P* = 0.008, 75% WL_max_ *P* = 0.001, 100% WL_max_ *P* < 0.001) and SV (25% WL_max_ *P* = 0.039, 75% WL_max_ *P* = 0.045, 100% WL_max_ *P* = 0.047) post‐LTx were also observed during incremental exercise to maximal exertion (Figure 2).

### 3.2 Leg muscle O_2_ consumption and microvascular oxygenation

Resting $P_{aO_{2}}$ and $S_{aO_{2}}$ did not change post‐LTx (pre‐LTx vs. post‐LTx $P_{aO_{2}}$, *P* = 0.177 and $S_{aO_{2}}$, *P* = 0.170; Table 2), but pre‐LTx, 8 of 10 patients received supplementary O_2_ (1–5 L min^−1^) via a nasal cannula, and none of the participants used supplementary O_2_ during the post‐LTx experiments. Despite the O_2_ supplementation, $P_{aO_{2}}$ decreased during all exercise intensities pre‐LTx (unloaded *P* < 0.0001, 10% WL_peak_ *P* < 0.0001, 20% WL_peak_ *P* < 0.0001), whereas $P_{aO_{2}}$ was maintained at resting levels during exercise post‐LTx (unloaded *P* = 0.894, 10% WL_peak_ *P* = 1.000, 20% WL_peak_ *P* = 1.000), significantly higher than pre‐LTx (unloaded *P* = 0.002, 10% WL_peak_ *P* < 0.001, 20% WL_peak_ *P* < 0.001; Table 2). Meanwhile, $P_{\bar{c}O_{2}}$ and $S_{\bar{c}O_{2}}$ were higher post‐LTx (pre‐LTx vs. post‐LTx $P_{\bar{c}O_{2}}$, *P* = 0.022 and $S_{\bar{c}O_{2}}$, *P* = 0.046). The $P_{\bar{c}O_{2}}$ was higher both at rest (*P* = 0.033) and during exercise (10% WL_peak_ *P* = 0.049 and 20% WL_peak_ *P* = 0.033). The $\mathrm{De}l_{O_{2}}$, $\mathrm{av}D_{O_{2}}$ or ${\dot{V}}_{O_{2}\mathrm{sm}}$ were not different pre‐ to post‐LTx at rest or during exercise (Table 2).

**TABLE 2:** Blood gases, skeletal muscle oxygen uptake and microvascular oxygenation at rest and during knee‐extension exercise when unloaded (0 W) and at 10% WL_peak_ and 20% WL_peak_.

Parameter	Pre LTx (waiting list)							Post LTx (6 months follow‐up)									
	Rest	Exercise 0 W		Exercise 10% WL_peak_		Exercise 20% WL_peak_		Rest	Exercise 0 W		Exercise 10% WL_peak_		Exercise 20% WL_peak_		Exercise	LTx	Exercise × LTX
		2 min	4 min	2 min	4 min	2 min	4 min		2 min	4 min	2 min	4 min	2 min	4 min	*P*‐value	*P*‐value	*P*‐value
$P_{aO_{2}}$, kPa	11.4 ±2.5	9.2 ±1.7	9.04 ±1.7	9.0 ±1.4	9.0 ±1.6	8.8 ±19	8.8 ±1.7	12.7 ±1.6	12.2 ±1.3	12.1 ±0.9	11.8 ±1.2	12.5 ±1.2	12.2 ±1.4	12.6 ±0.8	<0.001	0.008	0.015
$P_{vO_{2}}$, kPa	4.8 ±0.9	3.3 ±0.5	3.4 ±0.5	3.1 ±0.3	3.3 ±0.6	3.1 ±0.3	3.2 ±0.4	5.7 ±0.7	4.7 ±3.2	3.4 ±0.4	3.1 ±0.4	3.2 ±0.3	3.1 ±0.2	3.2 ±0.3	0.003	0.078	0.235
Hb, mmol L^−1^	9.1 ±1.2	9.0 ±1.4	9.0 ±1.5	8.7 ±1.4	9.0 ±1.5	9.2 ±1.3	9.0 ±1.5	7.4 ±0.7	7.4 ±0.9	7.5 ±0.9	7.3 ±0.7	7.3 ±0.6	7.3 ±0.8	7.6 ±0.3	0.605	0.027	0.559
$S_{aO_{2}}$, %	94 ±5	92 ±5	92 ±5	92 ±4	92 ±4	92 ±4	92 ±5	97 ±1	97 ±1	97 ±1	97 ±1	97 ±1	97 ±1	98 ±1	0.488	0.031	0.712
$S_{vO_{2}}$, %	63 ±15	34 ±6	36 ±7	32 ±4	33 ±5	31 ±5	31 ±6	75 ±6	43 ±24	35 ±5	32 ±5	33 ±4	32 ±4	32 ±4	<0.001	0.266	0.272
Venous lactate, mmol L^−1^	0.73 ±0.36	1.39 ±0.64	1.80 ±0.80	1.36 ±0.71	1.80 ±0.97	1.58 ±0.87	2.00 ±1.06	0.96 ±0.49	1.24 ±0.19	1.33 ±0.26	1.36 ±0.64	1.56 ±0.80	1.33 ±0.60	1.50 ±0.54	0.006	0.682	0.360
pH	7.41 ±0.03	7.40 ±0.04	7.39 ±0.04	7.41 ±0.03	7.39 ±0.03	7.40 ±0.04	7.39 ±0.04	7.40 ±0.05	7.40 ±0.04	7.39 ±0.04	7.41 ±0.04	7.40 ±0.04	7.40 ±0.02	7.39 ±0.04	0.265	0.972	0.729
$C_{aO_{2}}$, mmol L^−1^	8.5 ±1.1	8.3 ±1.1	8.4 ±1.2	8.1 ±1.3	8.3 ±1.2	8.5 ±1.2	8.3 ±1.2	7.1 ±0.6	7.3 ±0.8	7.4 ±0.9	7.2 ±0.7	7.3 ±0.6^†^	7.2 ±0.7	7.5 ±0.3	0.762	0.098	0.434
$C_{vO_{2}}$, mmol L^−1^	5.8 ±1.4	3.2 ±0.7	3.3 ±0.8	2.9 ±0.5	3.1 ±0.7	3.0 ±0.5	3.0 ±0.6	5.2 ±0.6	3.2 ±2.3	2.2 ±1.0	2.3 ±0.4	2.3 ±0.3	2.3 ±0.3	2.4 ±0.3	<0.001	0.114	0.542
$C_{cO_{2}}$, mmol L^−1^	7.0 ±1.0	5.7 ±0.7	5.7 ±0.7	5.5 ±0.8	5.6 ±0.8	5.7 ±0.7	5.6 ±0.7	5.6 ±1.6	5.5 ±1.6	5.0 ±0.5	4.8 ±0.4	4.8 ±0.3	4.8 ±0.4	5.0 ±0.2	<0.001	0.087	0.473
$P_{\bar{c}O_{2}}$, kPa	6.8 ±0.6	5.1 ±0.4	5.2 ±05	5.0 ±0.2	5.0 ±0.3	4.9 ±0.3	5.0 ±0.3	7.8 ±0.6	5.5 ±0.5	5.6 ±0.4	5.4 ±0.5	5.5 ±0.3	5.4 ±0.3	5.4 ±0.3	<0.001	0.022	0.240
$S_{\bar{c}O_{2}}$, %	78 ±8	64 ±3	63 ±3	63 ±2	62 ±3	61 ±2	63 ±3	86 ±4	66 ±4	66 ±2	65 ±3	65 ±2	65 ±3	65 ±2	<0.001	0.046	0.323
$\mathrm{De}l_{O_{2}}$, mmol min^−1^	2.15 ±1.29	6.94 ±2.19	7.89 ±1.78	7.19 ±1.49	8.56 ±2.15	7.76 ±1.15	8.14 ±1.32	2.61 ±0.94	5.93 ±2.59	5.63 ±1.80	6.31 ±1.44	6.83 ±2.14	5.95 ±1.61	6.50 ±1.37	<0.001	0.182	0.211
$C_{\left(a-v\right)O_{2}}$, mM	2.94 ±1.57	5.24 ±1.11	5.23 ±1.29	5.29 ±1.17	5.37 ±0.99	5.63 ±1.23	5.36 ±1.22	1.72 ±0.53	4.80 ±0.89	4.75 ±0.77	4.85 ±0.77	4.91 ±0.65	4.87 ±0.80	5.11 ±0.49	<0.001	0.324	0.456
EO_2_, ratio	0.34 ±0.16	0.63 ±0.08	0.62 ±0.09	0.65 ±0.06	0.65 ±0.06	0.66 ±0.07	0.64 ±0.07	0.24 ±0.07	0.65 ±0.07	0.64 ±0.04	0.67 ±0.06	0.67 ±0.05	0.67 ±0.05	0.68 ±0.05	<0.001	0.918	0.014
${\dot{V}}_{O_{2}\mathrm{sm}}$, mmol O_2_ min^−1^	0.8 ±0.6	4.3 ±1.2	4.9 ±1.5	4.6 ±0.9	5.5 ±1.1	5.1 ±0.6	5.4 ±1.5	0.6 ±0.2	3.9 ±2.0	3.6 ±1.3	4.2 ±1.0	4.6 ±1.6	4.0 ±1.2	4.7 ±1.2	<0.001	0.277	0.749
$D_{\mathrm{sm}O_{2}}$, mmol min^−1^ kPa^−1^	0.11 ±0.09	0.85 ±0.27	0.97 ±0.14	0.94 ±0.18	1.09 ±0.24	1.04 ±0.15	1.11 ±0.35	0.08 ±0.04	0.73 ±0.42	0.65 ±0.28	0.80 ±0.24	0.86 ±0.32	0.75 ±0.23	0.88 ±0.28	<0.001	0.178	0.574

*Note*: Data are expressed as the mean ± SD.

Abbreviations: $C_{\left(a-v\right)O_{2}}$, arterial‐to‐femoral venous O_2_ concentration difference; $C_{aO_{2}}$, arterial oxygen concentration; $C_{cO_{2}}$, capillary oxygen concentration; $C_{vO_{2}}$, venous oxygen concentration; $\mathrm{De}l_{O_{2}}$, O_2_ delivery; $D_{\mathrm{sm}O_{2}}$, skeletal muscle oxygen conductance; EO_2_, fractional O_2_ extraction; Hb, haemoglobin; LTx, lung transplantation; $P_{aO_{2}}$, arterial oxygen partial pressure; $P_{\bar{c}O_{2}}$, capillary O_2_ tension; $P_{vO_{2}}$, venous oxygen partial pressure; $S_{aO_{2}}$, arterial oxygen saturation; $S_{\bar{c}O_{2}}$, capillary O_2_ saturation; $S_{vO_{2}}$, venous oxygen saturation; ${\dot{V}}_{O_{2}\mathrm{sm}}$, skeletal muscle O_2_ uptake; WL_peak_, peak workload during one‐leg knee‐extension exercise.

Comparisons were made with linear mixed models and Tukey's *post hoc* test. Pre‐LTx *n* = 7, post‐LTx *n* = 7.

### 3.3 Leg muscle O_2_ conductance

Resting $D_{\mathrm{sm}O_{2}}$ and was similar pre‐LTx and post‐LTx (*P* = 0.431; Table 2). During exercise, $D_{\mathrm{sm}O_{2}}$ increased to similar values pre‐LTx and post‐LTx (unloaded *P* = 0.120; 10% WL_peak_ *P* = 0.209; 20% WL_peak_ *P* = 0.253). No difference was seen in EO_2_, either at rest (*P* = 0.189) or during exercise (unloaded *P* = 0.627; 10% WL_peak_ *P* = 0.395; 20% WL_peak_ *P* = 0.340), where EO_2_ increased to similar values pre‐LTx and post‐LTx (unloaded *P* = 0.627; 10% WL_peak_ *P* = 0.395; 20% WL_peak_ *P* = 0.340; Table 2).

### 3.4 Plasma NO_x_, 6‐keto‐PGF_1α_, 8‐isoprostane and pro‐BNP at rest and during KEE

**FIGURE 3:**
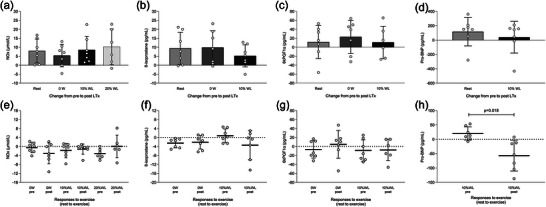
Plasma concentrations from the femoral vein of nitrite/nitrate (NOx), 8‐isoprostane, 6‐keto‐prostaglandin F1α (6kPGF1α) and pro‐brain natriuretic peptide (pro‐BNP) at rest and during submaximal one‐leg knee‐extensor exercise. (a) Changes in plasma NOx concentration from pre to post LTx. (b) Changes in plasma 8‐isoprostane concentration from pre to post LTx. (c) Changes in plasma 6kPGF1α concentration from pre to post LTx. (d) Changes in plasma pro‐BNP concentration from pre to post LTx. (e) Responses to exercise in plasma NOx concentration. (f) Responses to exercise in plasma 8‐isoprostane concentration. (g) Responses to exercise in plasma 6kPGF1α concentration. (h) Responses to exercise in plasma pro‐BNP concentration. Data are expressed as the mean ± SD before and after lung transplantation (LTx). Student's paired *t*‐test was used to explore differences between pre‐LTx and post‐LTx in response to exercise. A one‐way ANOVA was used to explore differences between rest and exercise (0 W, 10% of peak workload during one‐leg knee‐extension exercise (WLpeak) and 20% WLpeak.

There was no difference in changes from pre‐ to post‐LTX in exercising plasma NO_x_ (unloaded *P* = 0.471; 10% WL_peak_ *P* = 0.875; 20% WL_peak_ *P* = 0.627), 6‐keto‐PGF1α (unloaded *P* = 0.793; 10% WL_peak_ *P* = 0.999), 8‐isoprostane (unloaded *P* = 0.995; 10% WL_peak_ *P* = 0.617) or mean pro‐BNP concentrations (rest, pre‐LTx 143 ± 236 vs. post‐LTx 260 ± 122 pg mL^−1^, *P* = 0.168; 10% WL_peak_, pre‐LTx 163 ± 249 vs. post‐LTx 203 ± 83 pg mL^−1^, *P* = 0.651; Figure 3d). In response to exercise, there were no differences between pre‐LTx and post‐LTx in plasma NO_x_ (unloaded *P* = 0.174; 10% WL_peak_ *P* = 0.730; 20% WL_peak_ *P* = 0.268), 8‐isoprostane (unloaded *P* = 0.749; 10% WL_peak_ *P* = 0.091) or 6‐keto‐PGF_1α_ (unloaded *P* = 0.327; 10% WL_peak_ *P* = 0.913) concentrations (Figure 3). In response to exercise, plasma pro‐BNP concentration was lower post‐LTx compared with pre‐LTx (10% WL_peak_ *P* = 0.018; Figure 3h).

## 4 DISCUSSION

We studied the adaptation from end‐stage COPD to near normalisation of lung function at 6 months post‐LTx by assessing the integrative cardiovascular response to dynamic quadriceps muscle exercise. Despite augmented central haemodynamic responses to exercise post‐LTx, such as higher CO and SV and lower circulating pro‐BNP, LTx had no impact on any of our outcomes for peripheral muscle haemodynamic control, including no change in ${\dot{Q}}_{\mathrm{leg}}$ or muscle O_2_ conductance.

### 4.1 Exercising muscle hypoperfusion

In COPD patients, ranging from moderate to very severe, it has been documented that exercising ${\dot{Q}}_{\mathrm{leg}}$ is attenuated compared with healthy individuals (Brønstad et al., 2012; Hartmann et al., 2022; Iepsen et al., 2017; Richardson et al., 2004), but we found no change in ${\dot{Q}}_{\mathrm{leg}}$ during KEE post‐LTx. The low vasodilatory capacity during exercise might be explained to some extent by impaired local release of NO and prostacyclin in COPD (Iepsen et al., 2017), but neither of these compounds was upregulated post‐LTx, and we found no response to muscle contractions during KEE, which supports the unaltered muscle perfusion.

Oxygen delivery to skeletal muscle mitochondria is a major determinant of peripheral exercise capacity that depends on $C_{aO_{2}}$, blood flow and tissue capacity to extract O_2_ in the microcirculation, which rely on muscle capillarisation, the capillary to mitochondrial $P_{O_{2}}$ difference and mitochondrial oxidative capacity (Hepple et al., 2000; Poole et al., 2022). Our COPD patients exhibited lower muscle oxygenation during exercise pre‐LTx and, as expected, both microvascular ($P_{\bar{c}O_{2}}$ and $S_{\bar{c}O_{2}}$) and macrovascular oxygenation ($P_{aO_{2}}$) were higher during exercise post‐LTx, but with little or no impact on O_2_ delivery to the working muscle or related muscular adaptations that we assessed, such as leg lean mass or one‐leg WL_peak_. Likewise, $D_{\mathrm{sm}O_{2}}$ remained unaffected in the working muscle post‐LTx; this might be attributable, in part, to the low workloads during KEE that we applied. In fact, we found no change in ${\dot{V}}_{O_{2}\mathrm{sm}}$ when workloads were increased, which suggests that the difference between workloads (0 W, 10% and 20% of WL_peak_) were too small to be detected within group. The main question of the present study was, however, whether ${\dot{Q}}_{\mathrm{leg}}$ adapted from pre‐ to post‐LTx at the same relative workloads, because previous observations have suggested that the exercising muscle is hypoperfused in COPD patients compared with healthy subjects, despite similar ${\dot{V}}_{O_{2}\mathrm{sm}}$ (Hartmann et al., 2025; Iepsen et al., 2017). There exists limited information on muscle adaptations in clinical populations exposed to chronic tissue hypoxia, but healthy individuals exposed to high‐altitude‐induced hypoxaemia show loss of muscle mass and reduced exercising muscle perfusion despite acclimatisation (Calbet et al., 2003; Hansen et al., 2022). Chronic muscle tissue hypoxia has also been proposed to be driving the muscle pathologies in skeletal muscle in COPD (Attaway et al., 2024; Gosker et al., 2007). We did not observe any muscular changes 6 months post‐LTx, although macro‐ and microvascular oxygenation was higher. Thus, reversal of hypoxaemia is likely to be beneficial to increase exercise capacity in COPD but might not be a prerequisite for muscle adaptations.

Although exercising ${\dot{Q}}_{\mathrm{leg}}$ was unchanged post‐LTx, we found that SV and CO were higher during KEE post‐LTx, whereas MAP was lower. The fact that muscle perfusion was unaltered, whereas CO responses were higher during exercise, would suggest increased blood flow distribution to non‐exercising tissues (Vogiatzis et al., 2010). An explanation for this might be that the recipients might no longer experience hyperinflation in response to exercise, which increases pulmonary blood volume post‐LTx (Hartmann et al., 2024), owing to less mechanical compression of the large thoracic vessels and right ventricle (Smith et al., 2019). Interestingly, we also observed lower pro‐BNP responses to exercise post‐LTx, suggesting a reduction in myocardial wall stress owing to decreased ventricular stretch and pressure. This finding supports the notion of improved cardiac efficiency post‐LTx, where the heart can sustain higher output with less neurohormonal activation and mechanical strain. Nevertheless, it is likely that the central haemodynamic adaptations were favourable to increasing whole‐body exercise capacity, such as walking distance and physical activity during daily life. However, these improvements might not translate into enhanced local muscle endurance (WL_peak_), which remains limited by peripheral factors, such as reduced vasodilatory capacity, potentially linked to endothelial dysfunction, oxidative stress or impaired vascular responsiveness to exercise stimuli (Iepsen et al., 2017). Although both the muscle and vasculature might still be targeted by specific therapeutic interventions (Broxterman et al., 2021; Hartmann et al., 2025; Iepsen et al., 2024), development within this area might not focus exclusively on normalising the consequences of COPD, such poor oxygenation or declining daily physical activity.

### 4.2 Clinical implications

The hypoperfusion of contracting leg muscles might be COPD disease specific (Hartmann et al., 2022) and emphasises the systemic nature of the disease, in whuch the muscle vasculature seems especially affected. The vasculature shows a degree of plasticity to both pharmacological (Iepsen et al., 2024; Rossman et al., 2015) and non‐pharmacological interventions, such as exercise training (Broxterman et al., 2021; Hartmann et al., 2025). Here, recent data suggest a more personalised strategy, in which small‐muscle training might increase muscle O_2_ diffusion capacity in severe COPD by allowing a higher muscle mechanical and metabolic stimulus with a lower demand for CO and ventilation (Broxterman et al., 2021), whereas high‐intensity whole‐body exercise training has the potential of restoring blood flow to the exercising muscle in earlier stages of mild to moderate COPD (Hartmann et al., 2025), but it remains unknown whether muscle hypoperfusion is a cause or an effect of limb muscle dysfunction in COPD. The aetiology of limb muscle dysfunction in COPD has been linked to a decline in lung function and the accompanying physical inactivity (Gosker et al., 2007). The physiological rationale is seemingly logical; for example, loss of lung function lowers exercise capacity and physical activity level, which negatively impacts muscle function (Jaitovich & Barreiro, 2018; McDonald et al., 2014; van Bakel et al., 2021). It is, however, impossible to separate these two factors in the general population of patients with COPD, which is why we studied the transition from end‐stage COPD to LTx. Although COPD‐related muscle dysfunction might, to some extent, be reversed by pulmonary rehabilitation of 8–12 weeks duration, muscle vasculature adaptations to exercise training seem to be blunted in COPD compared with healthy individuals (Broxterman et al., 2021; Gouzi et al., 2013; Iepsen et al., 2016). Compared with adaptations to pulmonary rehabilitation, where 6MWD typically increases by 30–55 m (McCarthy et al., 2015), our cohort of LTx recipients showed a doubling of their daily physical activity level and 6MWD (∼200 m), but with no significant changes in leg muscle mass or function 6 months post‐LTx. Although physical inactivity might not be the main driver of muscle dysfunction in COPD, targeting the vasculature might improve peripheral exercise capacity.

For LTx recipients, a major concern is the immunosuppressive medications used in the post‐transplant period, which might worsen muscle function. Conversely, chronic systemic inflammation has been linked to loss of muscle mass (Tuttle et al., 2020). Corticosteroids have been shown to induce skeletal muscle myopathy, resulting in muscle fibre atrophy and connective tissue between fibres, leading to reduced force‐generating capacity and increased susceptibility to muscular fatigue (Maltais et al., 2014). In the present study, all LTx recipients received 5 mg of prednisolone day^−1^, but with a significantly higher dose in the initial weeks post‐LTx. Furthermore, our participants had been exposed to corticosteroids for years upon inclusion when treated for acute exacerbations of COPD. Animal studies have shown that the immunosuppressive agent cyclosporin, used by most of our LTx recipients, impairs exercise endurance by affecting mitochondrial oxidative capacity and respiration (Hokanson et al., 1995; Mercier et al., 1995). Previous results have suggested that inflammation does not impact muscle adaptations to exercise training in COPD (Ryrsø et al., 2018) or might even be a prerequisite for muscle regeneration (Rabinovich et al., 2003; Wedell‐Neergaard et al., 2019). Indeed, we cannot rule out that the immunosuppressants might have had a detrimental effect on skeletal muscle, which was balanced out by the beneficial systemic adaptations.

### 4.3 Limitations

There are some general limitations of this study to be considered. COPD is a heterogenetic disease, in which many patients experience limb muscle dysfunction; however, COPD patients who are eligible for LTx might not represent the general population. Despite higher physical activity levels and improved central exercise capacity and oxygenation post‐LTx, we cannot exclude the possibility that longer follow‐up (>6 months) would have induced muscle adaptations in LTx recipients. Likewise, more intensive rehabilitation programmes might have increased muscle function post‐LTx, but currently, data in favour of this strategy have not yet been convincing (Gutierrez‐Arias et al., 2021).

There are also some specific limitations of this study that must be considered here. First, the experimental protocol focused on submaximal one‐KEE, and measurements of ${\dot{Q}}_{\mathrm{leg}}$ during peak exercise were not obtained. This choice was deliberate, because submaximal workloads have previously been shown to be highly sensitive for detecting peripheral circulatory limitations (Hartmann et al., 2025; Iepsen et al., 2016, 2017, 2018). In these previous studies, the difference in exercising ${\dot{Q}}_{\mathrm{leg}}$ between COPD and healthy subjects was substantial (∼500 mL min^−1^; Iepsen et al., 2017), but responsive to exercise training (Hartmann et al., 2025; Iepsen et al., 2016), demonstrating that submaximal exercise (∼20% of WL_peak_) is a sensitive model for detecting peripheral circulatory limitations independent of central cardiopulmonary constraints.

Second, the study used a paired pre‐ versus post‐LTx design, without a non‐transplanted control group. Although this within‐subject comparison strengthens the ability to detect physiological changes over time, it also introduces potential confounding from factors that accompany transplantation but are not intrinsic to the transplant itself, most notably immunosuppressive therapy. As a result, it is not possible with certainty to disentangle effects attributable to LTx per se from those related to ancillary treatments or post‐transplant recovery processes. However, inclusion of a parallel non‐transplanted control group or randomisation to LTx versus no LTx is clearly neither feasible nor ethical in this population, and such confounding is therefore unavoidable in human studies of LTx. From a mechanistic perspective, one potential approach to address these limitations could be the incorporation of targeted exercise interventions, whether the observed dissociation between central and peripheral haemodynamic is reversible. However, implementing such interventions in LTx recipients presents substantial logistical and clinical challenges and was beyond the scope of the present study.

A further limitation of the present study relates to the use of supplementary O_2_ in a subset of participants before LTx. Although O_2_ was administered via nasal cannula and flow rates were prescribed on clinical grounds and kept consistent within individuals across testing sessions, this mode of delivery does not permit precise control of the fraction of inspired O_2_. Consequently, some interindividual variability in effective fraction of inspired O_2_ is likely to have occurred, which might have contributed to both random and systematic variability in measures of O_2_ transport. The magnitude of this effect cannot be quantified from the present data. Importantly, the use of supplementary O_2_ was clinically necessary to ensure participant safety and tolerability of the protocol and reflects real‐world conditions in this patient population, where contracting skeletal muscles are exposed to lower micro‐ and macrocirculatory levels of O_2_ before LTx. Nonetheless, variability in effective fraction of inspired O_2_ should be considered when interpreting the O_2_ transport measurements, particularly when comparing individuals requiring supplementary O_2_ with those breathing room air.

The assumption that mitochondrial $P_{O_{2}}$ approaches zero is reasonable during exercise, given that intracellular $P_{O_{2}}$ within the myocyte has been shown to decline rapidly with the onset of exercise and to stabilise at extremely low values (<0.5 kPa), even at submaximal intensities (Richardson et al., 1995), such that the precise value of mitochondrial $P_{O_{2}}$ has minimal mathematical influence on the derived $D_{\mathrm{sm}O_{2}}$ values (Table A1). At rest, however, this assumption is not necessarily entirely valid, given that intracellular $P_{O_{2}}$ in resting human skeletal muscle has been reported to be up to ∼4 kPa, implying that mitochondrial $P_{O_{2}}$ must also be clearly above zero, albeit lower than cytosolic values (Richardson et al., 2006; Wagner, 2012). The critical intracellular $P_{O_{2}}$ required to sustain mitochondrial ATP production at rest in human muscle in vivo has been estimated to be ∼0.35 kPa, providing a functional lower bound (Lanza et al., 2010). Regardless of its exact value, resting mitochondrial $P_{O_{2}}$ is expected to be higher than during exercise. Consequently, the assumption that mitochondrial $P_{O_{2}}$ is zero is likely to introduce some systematic bias into the resting estimates of $D_{\mathrm{sm}O_{2}}$, such that true resting values might be higher than those reported here, and the rest‐to‐exercise changes thus less pronounced.

Lastly, although an a priori power calculation indicated that a sample size of eight participants would be sufficient to detect a mean pre‐ to post‐LTx difference in ${\dot{Q}}_{\mathrm{leg}}$ of 300 mL min^−1^ during KEE, COPD patients on the waiting list for LTx remain a highly heterogeneous clinical population. Thus, the possibility of a type II error with respect to the reported absence of a detectable pre‐ to post‐LTx change in the ${\dot{Q}}_{\mathrm{leg}}$ response to KEE must be considered (Berg et al., 2024).

## 5 CONCLUSION

LTx did not affect peripheral haemodynamic responses to moderate‐intensity small muscle mass exercise or related muscular adaptations at 6 months follow‐up in patients with end‐stage COPD. Thus, the muscle blood flow regulation in the exercising leg remained unchanged by LTx. In contrast, central haemodynamics adapted favourably, with higher CO and SV and lower pro‐BNP responses to exercise after LTx. These findings highlight a persistent mismatch between central and peripheral haemodynamic adaptations to LTx. Future work might focus on the muscle vasculature to improve perfusion of exercising skeletal muscle in both stable COPD and LTx recipients, because impaired peripheral exercise capacity is a clinically significant challenge in these patients.

## AUTHOR CONTRIBUTIONS

Concept and design: Camilla Koch Ryrsø, Thomas Kromann Lund, Pia Thaning, Ronan M. G. Bergand and Ulrik Winning Iepsen. Acquisition, analysis or interpretation of data: All authors. Statistical analysis: Camilla Koch Ryrsø and Ulrik Winning Iepsen. Drafting of the manuscript: Camilla Koch Ryrsø and Ulrik Winning Iepsen. Critical revision of the manuscript for important intellectual content: All authors. Obtained funding: Camilla Koch Ryrsø and Ulrik Winning Iepsen. Administrative, technical or material support: Camilla Koch Ryrsø, Thomas Kromann Lund, Pia Thaning, Lasse Gliemann, Michael Perch, Jacob Peter Hartmann, Ida Kær Thorsen, Gregers Winding Munch, Michael Perch, Ronan M. G. Berg, Ylva Hellsten and Ulrik Winning Iepsen. Supervision: Thomas Kromann Lund, Pia Thaning, Lasse Gliemann, Michael Perch, Michael Perch, Ronan M. G. Berg and Ulrik Winning Iepsen. All authors approved the final version of the manuscript and agree to be accountable for all aspects of the work in ensuring that questions related to the accuracy or integrity of any part of the work are appropriately investigated and resolved. All persons designated as authors qualify for authorship, and all those who qualify for authorship are listed.

## CONFLICT OF INTEREST

The authors declare that the research was conducted in the absence of any commercial or financial relationships that could be construed as a potential conflict of interest.

## GENERATIVE AI STATEMENT

The authors confirm that no artificial intelligence tools, including large language models (LLMs), were used in the drafting or revision of this manuscript. All content was conceived, written and approved solely by the authors.

## Data Availability

Relevant anonymised participant data will be made available for others on a reasonable request to the corresponding author. The lead and corresponding authors affirm that the manuscript is an honest, accurate and transparent account of the study being reported; that no important aspects of the study have been omitted; and that any discrepancies from the study as planned and registered have been explained.

## 1

**TABLE A1:** *P*‐values from the linear mixed model comparing blood gases, skeletal muscle oxygen uptake and microvascular oxygenation at rest and during knee‐extension exercise when unloaded (0 W) and at 10% WL_peak_ and 20% WL_peak_.

Parameter	Effects of exercise						Effect of exercise						Effect of exercise × LTX						
	Pre LTx						Post LTx						Pre LTx vs. Post LTx						
	Rest vs. exercise 0 W		Rest vs. exercise 10% WL_peak_		Rest vs. exercise 20% WL_peak_		Rest vs. exercise 0 W		Rest vs. exercise 10% WL_peak_		Rest vs. exercise 20% WL_peak_		Rest	Exercise 0 W		Exercise 10% WL_peak_		Exercise 20% WL_peak_	
	2 min	4 min	2 min	4 min	2 min	4 min	2 min	4 min	2 min	4 min	2 min	4 min		2 min	4 min	2 min	4 min	2 min	4 min
	*P*‐value	*P*‐value	*P*‐value	*P*‐value	*P*‐value	*P*‐value	*P*‐value	*P*‐value	*P*‐value	*P*‐value	*P*‐value	*P*‐value	*P*‐value	*P*‐value	*P*‐value	*P*‐value	*P*‐value	*P*‐value	*P*‐value
$P_{aO_{2}}$, kPa	<0.001	<0.001	<0.001	<0.001	<0.001	<0.001	0.922	0.894	0.517	>0.999	0.859	>0.999	0.177	0.003	0.002	0.004	0.001	0.004	0.001
$P_{vO_{2}}$, kPa	0.029	0.045	0.006	0.090	0.013	0.005	0.981	0.001	<0.001	<0.001	0.001	<0.001	0.034	0.318	0.908	0.776	0.651	0.644	0.863
Hb, mmol L^−1^	>0.999	<0.999	0.982	>0.999	0.943	>0.999	0.570	0.673	0.455	0.027	0.077	0.675	0.004	0.040	0.049	0.054	0.034	0.023	0.079
$S_{aO_{2}}$, %	0.960	0.914	0.943	0.877	0.950	0.998	>0.999	>0.999	0.778	0.903	0.828	0.548	0.170	0.034	0.025	0.023	0.018	0.022	0.036
$S_{vO_{2}}$, %	0.006	0.008	0.002	0.002	<0.001	0.001	0.125	<0.001	<0.001	<0.001	<0.001	<0.001	0.073	0.386	0.833	0.941	0.952	0.899	0.744
Venous lactate, mmol L^−1^	0.217	0.112	0.373	0.197	0.492	0.268	0.515	0.645	0.307	0.281	0.033	0.0200	0.399	0.588	0.186	0.994	0.651	0.594	0.355
pH	0.998	0.733	>0.999	0.937	0.981	0.847	>0.999	0.994	>0.999	>0.999	>0.999	>0.999	0.766	0.960	0.947	0.867	0.883	0.829	0.892
$C_{aO_{2}}$, mmol L^−1^	0.999	>0.999	0.927	0.993	>0.999	0.999	0.613	0.706	0.564	0.049	0.038	0.621	0.017	0.101	0.129	0.145	0.085	0.076	0.181
$C_{vO_{2}}$, mmol L^−1^	0.002	0.002	0.001	<0.001	<0.001	0.001	0.322	<0.001	<0.001	<0.001	<0.001	<0.001	0.323	0.992	0.035	0.016	0.019	0.008	0.054
$C_{cO_{2}}$, mmol L^−1^	0.021	0.024	0.008	0.003	0.049	0.003	0.916	0.008	0.002	0.003	0.008	0.065	0.092	0.832	0.068	0.077	0.047	0.031	0.079
$P_{\bar{c}O_{2}}$, kPa	0.009	0.005	0.002	0.001	0.004	0.001	0.005	0.024	0.010	0.010	0.012	0.009	0.033	0.173	0.085	0.129	0.049	0.059	0.033
$S_{\bar{c}O_{2}}$, %	0.044	0.045	0.012	0.013	0.006	0.021	<0.001	<0.001	<0.001	<0.001	0.002	<0.001	0.069	0.236	0.067	0.208	0.078	0.122	0.173
$\mathrm{De}l_{O_{2}}$, mmol·min^−1^	0.004	0.003	0.001	<0.001	0.001	0.002	0.458	0.312	0.084	0.170	0.320	0.210	0.495	0.498	0.060	0.329	0.200	0.110	0.105
$C_{\left(a-v\right)O_{2}}$, mM	0.066	0.098	0.039	0.034	<0.001	0.002	<0.001	<0.001	0.001	0.001	0.008	<0.001	0.093	0.458	0.433	0.443	0.353	0.268	0.658
EO_2_, ratio	0.013	0.019	0.005	0.005	<0.001	0.001	<0.001	<0.001	<0.001	<0.001	0.002	<0.001	0.189	0.572	0.627	0.569	0.395	0.748	0.340
${\dot{V}}_{O_{2}\mathrm{sm}}$, mmol O_2_ min^−1^	0.005	0.005	<0.001	0.001	<0.001	0.002	0.147	0.057	0.016	0.048	0.082	0.018	0.528	0.735	0.151	0.470	0.336	0.176	0.402
$D_{\mathrm{sm}O_{2}}$, mmol min^−1^ kPa^−1^	0.005	0.009	<0.001	<0.001	<0.001	<0.001	0.167	0.077	0.025	0.050	0.065	0.024	0.431	0.610	0.120	0.315	0.209	0.083	0.253

*Note*: Comparisons were made with linear mixed models and Tukey's *post hoc* test. Pre‐LTx *n* = 7, post‐LTx *n* = 7.

Abbreviations: $C_{\left(a-v\right)O_{2}}$, arterial‐to‐femoral venous O_2_ concentration difference; $C_{aO_{2}}$, arterial oxygen concentration; $C_{cO_{2}}$, capillary oxygen concentration; $C_{vO_{2}}$, venous oxygen concentration; $\mathrm{De}l_{O_{2}}$, O_2_ delivery; $D_{\mathrm{sm}O_{2}}$, skeletal muscle oxygen conductance; EO_2_, fractional O_2_ extraction; Hb, haemoglobin; LTx, lung transplantation; $P_{aO_{2}}$, arterial oxygen partial pressure; $P_{\bar{c}O_{2}}$, capillary O_2_ tension; $P_{vO_{2}}$, venous oxygen partial pressure; $S_{aO_{2}}$, arterial oxygen saturation; $S_{\bar{c}O_{2}}$, capillary O_2_ saturation; $S_{vO_{2}}$, venous oxygen saturation; ${\dot{V}}_{O_{2}\mathrm{sm}}$, skeletal muscle O_2_ uptake; WL_peak_, peak workload during one‐leg knee‐extension exercise.
